# Obturator hernia: a case report

**DOI:** 10.1186/s13256-021-02793-7

**Published:** 2021-06-17

**Authors:** C. Kendall Major, Madiha Aziz, Jay Collins

**Affiliations:** Department of Surgery, Eastern Virginia Medical School, 825 Fairfax Avenue, Norfolk, VA USA

**Keywords:** Hernia, Obturator hernia, General surgery, Bowel obstruction, Laparoscopic, Case report

## Abstract

**Background:**

Obturator hernia is rare and accounts for less than 1% of all abdominal wall hernias. It represents a diagnostic challenge due to its nonspecific signs and symptoms.

**Case presentation:**

We present a case of an 89-year-old caucasian female with a 12-hour history of right medial thigh pain. Computed tomography scan revealed a right obturator hernia with small bowel obstruction. The hernia was successfully repaired laparoscopically without any need for small bowel resection. She was discharged on postoperative day 2 with an uneventful recovery and zero complications.

**Conclusion:**

This case report highlights the importance of rapid diagnosis and repair of obturator hernia even in the setting of an improving clinical picture. It also demonstrates the safety of laparoscopic repair in this setting.

## Background

Obturator hernias are rare and account for less than 1% of all abdominal wall hernias [1]. These hernias have a high mortality rate due to their nonspecific signs and symptoms during presentation [2]. Common signs and symptoms include symptoms of bowel obstruction such as nausea, vomiting, and abdominal pain, along with pain in the groin or medial thigh. Obturator hernias occur more often on the right side than the left side because the left side is covered by the sigmoid colon [3, 4]. They form most often in individuals with a low-normal or underweight body mass index (BMI). Here, we present a case where a timely diagnosis and prompt surgical repair of an obturator hernia led to an uneventful and uncomplicated postoperative course.

## Case presentation

An 89-year-old caucasian female presented to the emergency department at an outside hospital with a 12-hour history of right medial thigh pain that radiated to the right buttocks and down the right leg. She reports that she was in the attic that morning when she stepped over a box and felt a sharp pain in her groin. The patient also reported the appearance of small bulge in her groin that had disappeared prior to her presentation. The patient’s medical history was significant for atrial fibrillation and transient ischemic attack. She also had a history of breast cancer 30 years ago and is now cancer free. Current medications were significant for Eliquis 2.5 mg twice daily. Surgical history was significant for laparoscopic cholecystectomy, double mastectomy, and total abdominal hysterectomy. There was no pertinent family history relating to this case. She has had two pregnancies and has two living children, which were born via standard vaginal delivery. Socially, the patient is widowed, retired, lives at home, and does not smoke or drink alcohol.

**Fig. 1:**
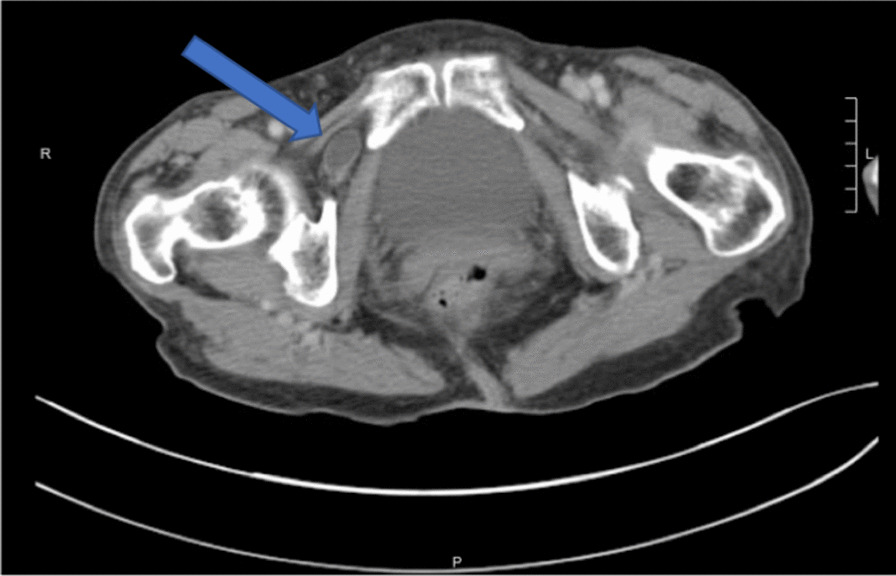
Axial computed tomography image showing a right-sided obturator hernia (denoted by the blue arrow)

**Fig. 2:**
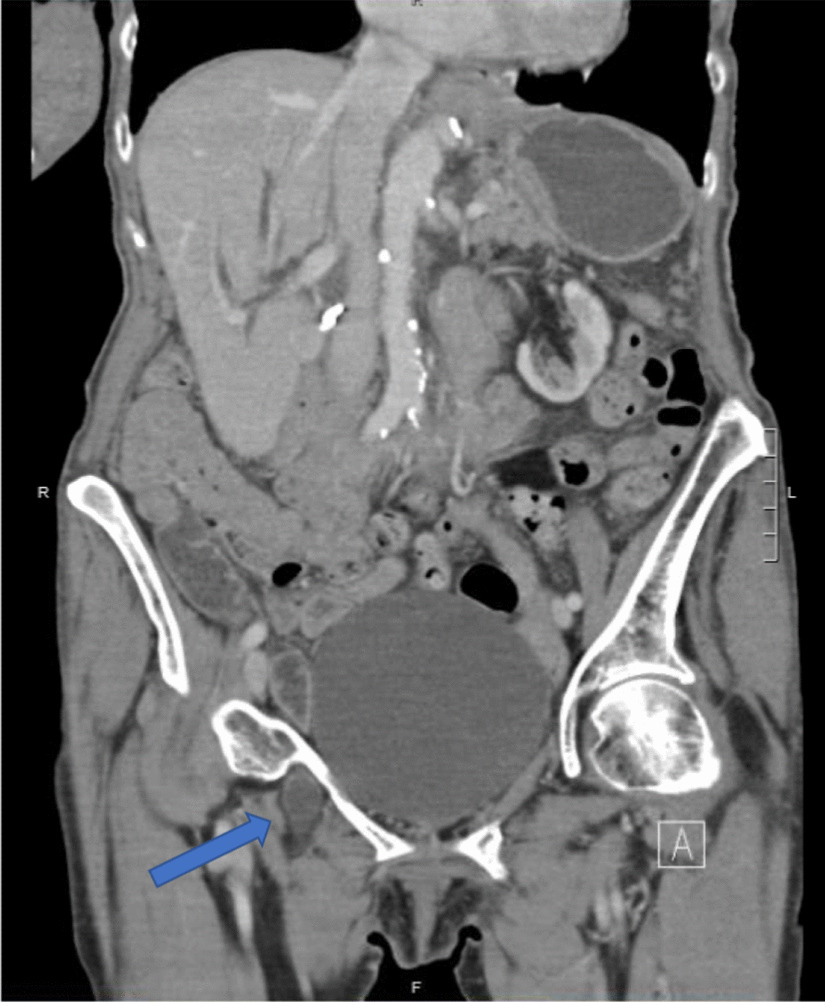
Coronal computed tomography scan showing a right-sided obturator hernia (denoted by the blue arrow)

Physical examination revealed a pleasant frail female in no acute distress. Her heart had a regular rate and rhythm, and her lungs were clear to auscultation bilaterally. She exhibited mild distention of the abdomen and no peritoneal signs. Neurologically, she was intact with no focal deficits; her sensation and strength were intact and equal on both sides. She had no lymphadenopathy, edema, or cyanosis. Blood pressure was 158/70, pulse was 80 with regular rate and rhythm, and temperature was 97.8℉. BMI was 16.46 kg/m^2^. Inguinal and femoral hernias were not found on light or deep palpation. Rectal examination revealed no frank blood, fissures, or palpable masses. Complete blood count, basic metabolic panel, hepatic function panel, magnesium, phosphorus, and prothrombin time and international normalized ratio (PT-INR) were all within normal limits. CT scan of the abdomen and pelvis identified a loop of small bowel in the right obturator canal that appeared to be causing a small bowel obstruction (Figs.1 and 2).

**Fig. 3:**
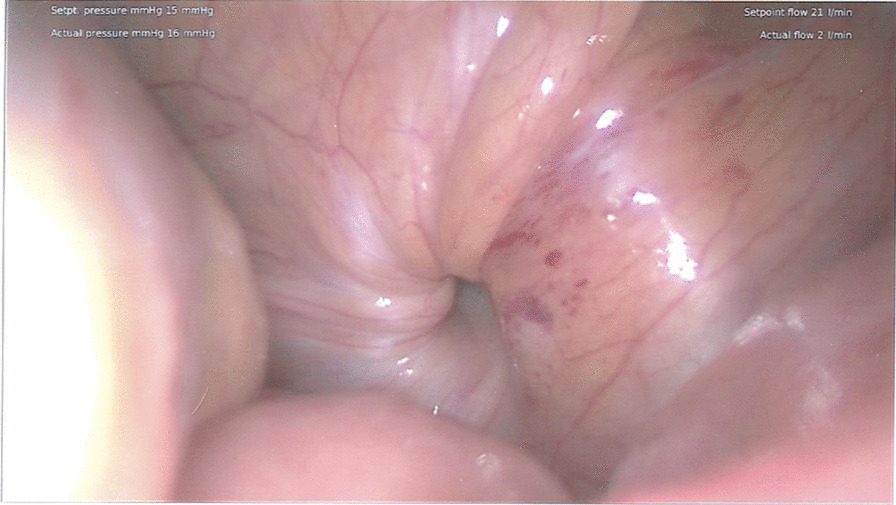
Dilated loops of bowel surrounding the obturator hernia after reduction

Patient was made nil by mouth, given Rocephin 1 g intravenous and Flagyl 500 mg intravenous, and taken to the operating room emergently for diagnostic laparoscopy with transabdominal extraperitoneal obturator hernia repair, and possible small bowel resection. On initial laparoscopic exploration, several loops of dilated bowel were visualized. A loop of bowel was found within the right obturator canal, was reduced, and displayed an obvious transition point. The bowel was pink and appeared healthy; therefore, resection was not performed (Fig. 3). A flap of peritoneum was dissected back to expose the pelvic floor and obturator hernia. A piece of polypropylene mesh was placed over the entire inguinal floor to include the obturator foramen. The pelvic floor was reperitonealized. Postoperatively, she resumed her normal diet without nausea or vomiting and began passing flatus. She was discharged on postoperative day 2 without any complications with five tablets of Roxicodone PO 5 mg for pain control. Because of the out-of-town location of the patient’s home address, further follow-up was not possible.

## Discussion

This case describes an elderly female with an incarcerated obturator hernia who was successfully treated with laparoscopic hernia repair. Although our patient appeared to have an improving clinical picture, prompt operative intervention was required to prevent the morbidity of ischemia or bowel perforation. Due to the rarity of obturator hernias, there is a paucity of literature on successful laparoscopic treatment in the setting of bowel incarceration. Additionally, her use of anticoagulation for atrial fibrillation also presented a unique challenge in the setting of emergency surgery.

Obturator hernias are exceedingly rare and can be difficult to diagnose [1, 5]. They are more common in women because of women having a broader pelvis. Risk factors for obturator hernia include advanced age, low body mass index, and multiparity. Due to the nonspecific signs and symptoms, timely diagnosis of obturator hernia can be challenging. CT scan can be especially useful in cases when physical examination is unrevealing or nonspecific [6, 7]. Diagnostic laparoscopy can also serve as a tool during emergency repair by which to detect occult or missed hernias [8].

There are two physical examination maneuvers that can be used if there is suspicion for obturator hernia: Howship–Romberg sign and Hannington-Kiff sign. The Howship–Romberg sign presents with inner thigh pain that is worsened by adduction, extension, and medial rotation of the thigh. This is due to compression of the cutaneous branch of the obturator nerve. This sign, however, is estimated to be present in less than 50% of cases [9]. The Hannington-Kiff sign occurs when there an intact patellar reflex with a concurrent loss of the thigh adductor reflex. This is due to obturator nerve compression causing weakness of the adductor muscles. This sign is considered to be more specific; however, it is less commonly seen [9, 10]. These maneuvers were not performed on our patient since a CT scan had revealed obturator hernia. However, if a CT scan is not readily available, these physical examination maneuvers could aid in the diagnosis of obturator hernia.

Accurate diagnosis early in the clinical course is uncommon, and therefore obturator hernias have a high mortality rate [1]. The only treatment for obturator hernia is surgery. A laparoscopic approach may be more beneficial for patients, especially in the elderly, given that fewer postoperative complications are seen with laparoscopic repair [11, 12]. A retrospective study demonstrated that emergency laparoscopic groin hernia repair has a lower morbidity in comparison with open repair in the setting of acute strangulated groin hernias [13].

Due to early recognition and prompt surgical repair, our patient had an uncomplicated postoperative course and was discharged 2 days later. It is imperative that obturator hernias are recognized early to prevent morbidity and mortality.

## Conclusion

Obturator hernias are uncommon and difficult to diagnose. They occur most commonly in elderly women. If not diagnosed and treated in a timely manner, they may result in bowel ischemia, perforation, sepsis, and death. In the case of an elderly patient presenting with medial thigh pain and mild abdominal distention, obturator hernia should be high on the differential list because of the high morbidity and mortality if undiagnosed. Computed tomography of the abdomen and pelvis is critical to the timely diagnosis. We have demonstrated rapid diagnosis and intervention can lead to a favorable outcome.

## Data Availability

All of the data is available in this manuscript.

## Abbreviations

CT: Computerized tomography
BMI: Body mass index
